# The Sphingolipid Inhibitors Ceranib-2 and SKI-II Reduce Measles Virus Replication in Primary Human Lymphocytes: Effects on mTORC1 Downstream Signaling

**DOI:** 10.3389/fphys.2022.856143

**Published:** 2022-03-17

**Authors:** Janice Chithelen, Hannah Franke, Nora Länder, Anika Grafen, Jürgen Schneider-Schaulies

**Affiliations:** Institute for Virology and Immunobiology, University of Würzburg, Würzburg, Germany

**Keywords:** acid ceramidase inhibitor ceranib-2, sphingosine kinase inhibitor SKI-II, mTORC1, translation, measles virus

## Abstract

The bioactive sphingolipids ceramide and sphingosine-1-phosphate (S1P) are involved in the regulation of cell homeostasis and activity ranging from apoptosis to proliferation. We recently described that the two compounds ceranib-2 (inhibiting acid ceramidase) and SKI-II [inhibiting the sphingosine kinases 1 and − 2 (SphK1/2)] reduce mTORC1 activity and measles virus (MV) replication in human primary peripheral blood lymphocytes (PBL) by about one log step. We now further investigated whether mTORC1 downstream signaling and viral protein expression may be affected by ceranib-2 and/or SKI-II. Western blot analyses showed that in uninfected cells the phosphorylation of the eukaryotic initiation factor 4E (eIF4E) was reduced by both inhibitors. Interestingly, MV infection led to an increase of rpS6 protein levels and phosphorylation of eIF4E. Treatment with both inhibitors reduced the rpS6 protein expression, and in addition, SKI-II reduced rpS6 phosphorylation. The phosphorylation of eIF4E was slightly reduced by both inhibitors. In addition, SKI-II led to reduced levels of IKK in MV-infected cells. Both inhibitors reduced the expression of viral proteins and the titers of newly synthesized MV by approximately one log step. As expected, SKI-II and rapamycin reduced also the virally encoded GFP expression; however, ceranib-2 astonishingly led to increased levels of GFP fluorescence. Our findings suggest that the inhibitors ceranib-2 and SKI-II act *via* differential mechanisms on MV replication. The observed effects on mTORC1 downstream signaling, predominantly the reduction of rpS6 levels by both inhibitors, may affect the translational capacity of the cells and contribute to the antiviral effect in human primary PBL.

## Introduction

Bioactive sphingolipids are potent key regulators of the cell metabolism and therefore also influence the life cycles of viruses. Vice versa, viral infections affect the sphingolipid metabolism and associated signaling pathways. Ceramides of various chain lengths and sphingosine 1 phosphate (S1P) are the best investigated bioactive sphingolipids. Ceramides are produced by *de novo* synthesis and further processed to derivatives such as glycosylated forms and sphingomyelins, which are major components of cellular membranes. From these cellular stores, ceramides can be released quickly in response to various stimuli, such as membrane perturbation or ER stress, resulting in the activation of sphingomyelinases. Ceramides then interact with a number of ceramide binding proteins such as SET, which regulates the phosphatase PP2A, and protein kinases such as protein kinase C-ζ (PKCζ; Wang et al., 2005; Oaks and Ogretmen, 2014). They can also be cleaved by ceramidases to release fatty acids and sphingosine, which is further phosphorylated by sphingosine kinases 1 and 2 (SphK1/2) to S1P (Spiegel and Milstien, 2007). The signaling by S1P occurs intracellularly, or after release from the cell extracellularly *via* S1P-receptors (Spiegel and Milstien, 2011). Intracellularly, S1P interacts with TRAF2, RIP1, Hsp90, GRP94, and NOD1/2, RIP2 leading to activation of NF-kB and mTORC, linking the signal to cellular translation and survival (Alvarez et al., 2010; Park et al., 2015, 2016; Pei et al., 2021).

It has been reported that viruses can stimulate cells so that viral uptake or the intracellular milieu and replication are improved. For example, adenovirus induces calcium influx and lysosomal exocytosis, a membrane repair mechanism resulting in the release of acid sphingomyelinase (ASM) and degradation of sphingomyelin to ceramides in the plasma membrane, which increases the viral endocytosis (Luisoni et al., 2015). Similarly, MV induces surface expression of its receptor CD150 *via* DC-SIGN-mediated activation of sphingomyelinases in dendritic cells, and thereby stimulates its own uptake into these cells (Avota et al., 2011). We recently observed that MV infection induces a transient S1P increase which may stimulate their translational capacity and thus support viral replication (Grafen et al., 2019). Sphingosine kinase-1 has earlier been identified to support MV and influenza virus replication and the S1P-metabolizing enzyme lyase reduced influenza virus propagation and cytopathogenicity (Seo et al., 2010, 2013; Vijayan et al., 2014). For further information on the role of sphingolipids in viral infections, see Avota et al. (2021), Beckmann and Becker (2021), and Schneider-Schaulies et al. (2021). These findings highlight various interactions of viruses with the sphingolipid metabolism and point to potential sites of therapeutic interventions against viral infections.

Despite the availability of an effective vaccine, measles remains a leading cause of morbidity and mortality in young children causing approximately 100,000 deaths each year worldwide (WHO, 2019). Due to the ongoing SARS-coronavirus-2 pandemic causing COVID-19, it is expected that measles vaccination campaigns are ceased and the number of cases will rise (Roberts, 2020). Although promising efficacious inhibitors have been characterized (Cox et al., 2020; Wittwer et al., 2021), only limited treatment options such as the use of ribavirin are in place and an efficient antiviral therapy against acute or persistent MV infections including subacute sclerosing panencephalitis (SSPE) is not yet clinically available (Reuter and Schneider-Schaulies, 2010). Alternative treatment options supporting the available ones would be desirable.

It has been reported that inhibition of the sphingosine kinases-1 and -2 by the inhibitor SKI-II reduced MV replication in epithelial and lymphoid cell lines (Vijayan et al., 2014). We recently found that the compounds ceranib-2 and SKI-II reduced mTORC1 activity and MV replication in human primary PBL (Grafen et al., 2019). It is known that mTORC1 and the S6 kinase (S6K) regulate cap-dependent mRNA translation *via* eIF4E phosphorylation and ribosomal translation *via* ribosomal protein S6 (rpS6) phosphorylation. Both activities are required for the proliferation of cells after mitogenic stimulation. We therefore wanted to explore whether ceranib-2 and/or SKI-II may affect cap-dependent mRNA translation and/or 5′ terminal oligopyrimidine (5’TOP) RNA translation and ribosomal activity, both of which finally would affect viral replication. We investigated MNK1 and eIF4E phosphorylation as a measure of cap-dependent mRNA translation, and rpS6 phosphorylation as a measure of 5’ TOP containing mRNAs translation, and quantified viral protein levels in inhibitor treated infected and uninfected PBL.

## Materials and Methods

### Cells, Viruses, and Inhibitors

All experiments involving human cells were conducted according to the principles expressed in the Declaration of Helsinki and ethically approved by the Ethics Committee of the Medical Faculty of the University of Würzburg. Primary human peripheral blood mononuclear cells (PBMC) obtained from leuko-reduction chambers of thrombocyte donations of anonymous healthy adult volunteers were diluted 1:5 in Versene (Gibco), layered into Histopaque^®^ 1,077, and purified by density gradient centrifugation. Isolated PBMC were washed three times with Ca^++^- and Mg^++^-free PBS and suspended in RPMI 1640 (Gibco) medium containing 10% FBS and incubated for 2 h on plastic dishes to remove adherent monocytes. Peripheral blood lymphocytes (PBL) were collected and stimulated with phytohemagglutinin-L (PHA, 2.5 μg/ml, Roche). Stimulation was controlled by measuring CD69 and CD25 expression by flow cytometry.

The recombinant wild-type MV rMV_IC323_eGFP (Hashimoto et al., 2002) was propagated using Vero cells expressing CD150 (Vero-hSLAM) cells. To determine MV titers, virus was harvested at the indicated times by freezing and thawing the complete culture; thus, cell-associated and virus in the supernatant were harvested together, and titrated using Vero-hSLAM cells. The inhibitors of SphK-1 and -2, SKI-II (French et al., 2003), and of acid ceramidase, ceranib-2 (Draper et al., 2011), were purchased from Sigma-Aldrich and dissolved in DMSO. The viability of cells in the presence of inhibitors was determined after 48 h incubation by flow cytometry using propidium iodide (PI; Biolegend) staining for dead cells as described (Grafen et al., 2019).

### Antibodies and Flow Cytometry

The following primary antibodies were used in immunoblotting and flow cytometry: rabbit anti-GAPDH (Santacruz sc-25,778), rabbit anti-human CD69 APC-conjugated (Biolegend 310,910) and FITC-conjugated (Biolegend 310,904), rabbit anti-human CD25 FITC-conjugated (Immunotools 218,102,535), rabbit anti-phosphoEIF4E (CST-9741), rabbit anti-total EIF4E (CST-9742), rabbit anti-phospho MNK1 (ThermoFischer PA5-110137), rabbit anti-total MNK1 (CST-2195), rabbit anti-total rpS6 (Sigma SAB4502676), rabbit anti-phospho rpS6 (CST-4858), rabbit anti-total IKK-β (CST-2684), and rabbit anti-mouse IgG1k FITC-conjugated (BD Biosciences 55,066). The mouse monoclonal antibodies F227 against MV-nucleocapsid (MV-N) and L77 against MV-hemagglutinin (MV-H) were generated and purified in our laboratory. For flow cytometry, 3 × 10^4^ cells per sample were surface stained with respective antibodies in FACS buffer (PBS containing 0.4% BSA and 0.02% sodium azide). Dead cells were stained with propidium iodide (Biolegend). Cells were acquired immediately using a BD FACSCalibur and the data were analyzed using FlowJo (Cytek Development) software.

### SDS-PAGE and Immunoblotting

Cells (1 × 10^7^) were lysed at 4°C for 1 h in 100 μl ml of RIPA lysis buffer (EMD Millipore 10× buffer diluted to 1× in sterile distilled water) containing complete protease and phosphatase inhibitors cocktail (Roche 04693124001 and 04906837001, respectively) and 1 mm DL-dithiothreitol (DTT). The protein quantification was done using the bicinchoninic acid (BCA solution Sigma B9643) assay. An equal amount of proteins was heated at 96°C for 7 min in reducing 4× Laemmli buffer (50 mm Tris HCl pH 6.8, 2% SDS, 10% glycerol, 1% β-mercaptoethanol, 12.5 mm EDTA, and 0.02% bromophenol blue) and applied to 12% SDS poly acrylamide gel electrophoresis. Proteins were blotted semidry on nitrocellulose membranes (Amersham GE 10600001) followed by blocking with 5% dry milk (AppliChem) or 5% BSA in PBS or Tris buffered saline, respectively, with 0.05% Tween-20. The membranes were then incubated with specific primary antibodies and HRP-conjugated secondary antibodies. Signals were visualized using chemiluminescent FemtoMax^™^ super sensitive HRP substrate (Rockland). The densitometric quantification of protein bands of target proteins and respective housekeeping gene products were done using Li-Cor Odyssey Pc imaging System Image Studio Version 4.0 (Li-Cor Biosciences). The fold changes in target proteins were normalized to band densities of respective GAPDH and fold changes in phosphorylated proteins were normalized to the band densities of total protein or GAPDH levels. All Western blotting experiments were repeated at least three times and representative images are shown.

### Statistical Analysis

Statistical analysis was performed using Microsoft Excel or GraphPad. Analysis was done using paired one-tailed student’s *t*-test and more than two groups were analyzed with one-way ANOVA. Values of *p* ≤ 0.05 were considered statistically significant (^*^p ≤ 0.05, ^**^p ≤ 0.01, and ^***^p ≤ 0.001). The data represent mean ± SD of at least three independent experiments.

## Results

### Ceranib-2 and SKI-II Reduce the Phosphorylation of rpS6 and eIF4E in Uninfected PBL

**Figure 1:**
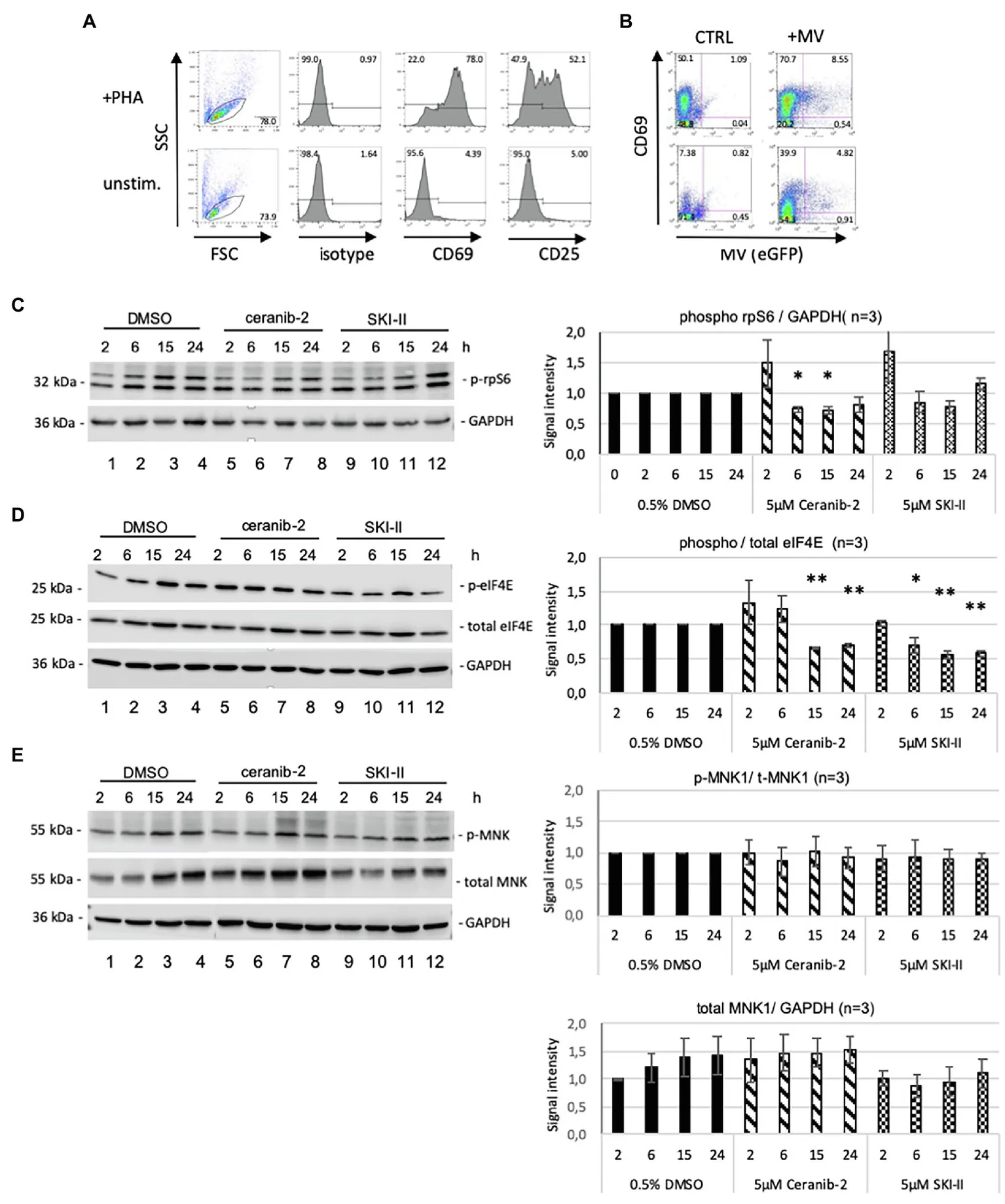
Effects of ceranib-2 and SKI-II on rpS6, eIF4E, and MNK1 in PBL. In all experiments, the quality of the PBL after PHA (2.5 μg/ml) stimulation was controlled by flow cytometry measuring the expression of CD69 and CD25 **(A)**, and the infection with MV (MV IC323eGFP) measuring the expression virally encoded GFP **(B)**. An example of infected and uninfected PBL in the presence and absence of PHA is shown representatively (data from Grafen et al., 2019 were reused). In **(C)**, Western blotting was performed to detect phosphorylated rpS6 and GAPDH. The corresponding quantification of the blot is shown on the right side (*n* = 3; blots from one representative experiment are shown). The reduction of rpS6 phosphorylation by ceranib-2 at 6 and 15 h was significant in comparison with corresponding time points of control signals (with ^*^p ≤ 0.05 calculated by student’s *t*-test). In **(D)**, Western blotting was performed to detect phosphorylated eIF4E, total eIF4E, and GAPDH. The quantification with normalization to the corresponding DMSO control values for each time point is shown on the right side. The values for phosphorylated over total eIF4E signals are given. Significances were calculated by student’s *t*-test by comparing corresponding time points of inhibitor treated with control signals (*n* = 3; with ^*^p ≤ 0.05 and ^**^p ≤ 0.01). In **(E)**, Western blotting was performed to detect phosphorylated MNK1, total MNK1, and GAPDH. Quantifications of the phosphorylated over total MNK1 and total MNK1 protein over GAPDH are given. Some blots were used for detection of two different proteins (MNK1 and eIF4E) and GAPDH controls were used for both. In some cases, two blots with lysates from the same experiment were used for the detection of total and phosphorylated proteins, and the corresponding GAPDH controls were used for quantification. In this case, only one representative GAPDH control is shown.

PBL from healthy donors were stimulated with PHA for 24 h prior to treatment with inhibitors. The surface expression of activation markers CD69 and CD25 (Figure 1A) and the viability of cells (propidium iodide staining, not shown) were checked in all experiments by flow cytometry. In the case of MV infection, the virally encoded GFP was measured as a control of infection (Figure 1B).

To determine the effects of ceranib-2 and SKI-II, PHA-stimulated PBL were treated for indicated times with 5 μm ceranib-2 and SKI-II and processed for Western blotting. As mTORC1 activity regulates pathways affecting translation, we probed for rpS6 and eIF4E phosphorylation. The rpS6 phosphorylation was moderately reduced by ceranib-2 at 16 h post-treatment (Figure 1C). A tendency of decreased rpS6 phosphorylation was also observed after treatment of cells for 6 and 15 h with SKI-II. The eIF4E phosphorylation was reduced in the PBL at 15 and 24 h after treatment with both inhibitors (Figure 1D).

In addition, we probed for MNK1 which is associated with the mTORC1 and can also regulate translation (Batool et al., 2020). The phosphorylation of MNK1 was not altered by treatment of the cells with the inhibitors (Figure 1E). However, we observed that total MNK1 expression levels increased over time in untreated and ceranib-2 treated cells, and that in the presence of SKI-II, there was a tendency to keep the MNK1 levels low (Figure 1E, lower panel).

Thus, treatment with ceranib-2 and SKI-II mainly affected the phosphorylation of eIF4E and moderately that of rpS6 in uninfected PBL. This led us to explore whether similar effects can also be observed in MV-infected PBL or whether the infection may alter the findings.

### Effects of SKI-II and Ceranib-2 in MV-Infected PBL

**Figure 2:**
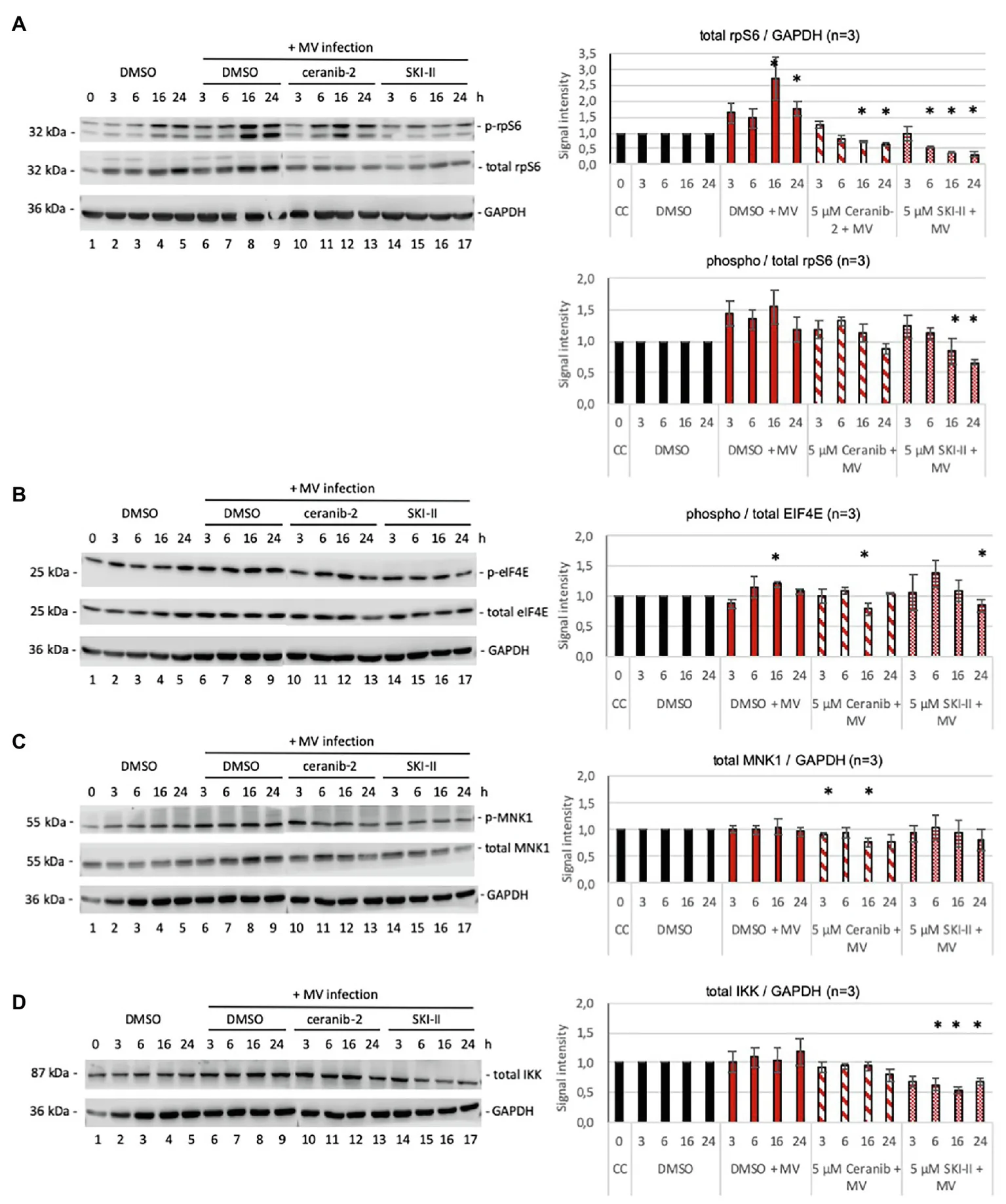
Effects of ceranib-2 and SKI-II on rpS6, eIF4E, MNK1, and IKK in MV-infected PBL. In **(A)**, Western blotting was performed to detect phosphorylated rpS6, total rpS6 protein, and GAPDH. The quantification with normalization to the corresponding DMSO control values for each time point is shown on the right side. The values for phosphorylated over total rpS6 and total rpS6 protein over GAPDH signals are given. The increase of total rpS6 in comparison with the DMSO control was significant at 16 and 24 h after infection. The 16 and 24 h values of ceranib-2-treated infected cells and the values of 6, 16, and 24 h SKI-II-treated infected cells were significantly reduced in comparison with both, uninfected and infected cell values. The phosphorylation of rpS6 was significantly reduced by SKI-II at 16 and 24 h in comparison with infected cell values (*n* = 3; with ^*^p ≤ 0.05). In **(B)**, Western blotting was performed to detect phosphorylated eIF4E, total eIF4E protein, and GAPDH. The quantification with normalization to the corresponding DMSO control values for each time point is shown on the right side. The values for phosphorylated over total eIF4E signals are given. Significances were calculated by student’s *t*-test by comparing corresponding time points of inhibitor treated with control signals (*n* = 3; with ^*^p ≤ 0.05). In **(C)**, Western blotting was performed to detect phosphorylated MNK1, total MNK1 protein, and GAPDH. Only the values for total MNK1 over GAPDH signals are given, because there were no significant differences in the phosphorylation of MNK1. Significances were calculated by student’s *t*-test by comparing corresponding time points of inhibitor treated with control signals (*n* = 3; with ^*^p ≤ 0.05). In **(D)**, Western blotting was performed to detect total IKK protein and GAPDH. The quantification with normalization to the corresponding DMSO control values for each time point is shown on the right side. The values for total IKK over GAPDH signals are given. Significances were calculated by student’s *t*-test by comparing corresponding time points of inhibitor treated with control signals (*n* = 3; with ^*^p ≤ 0.05). Some blots were used for detection of two different proteins (MNK1 and IKK) and GAPDH controls were re-used, and in some cases, two blots with lysates from the same experiment were used for the detection of total and phosphorylated proteins. The corresponding GAPDH controls were used for quantification. Only blots from one representative experiment are shown.

PHA-stimulated PBL were infected with recombinant wild-type MV IC323-eGFP at a MOI of 1 in the absence and presence of 5 μm ceranib-2 and SKI-II, and were cultivated uninfected in the presence of DMSO as control. After 3, 6, 16, and 24 h, cells were then processed for Western blotting. The MV infection stimulated the expression of total rpS6 in comparison with control treatment (Figure 2A, lanes 8, 9 compared to lanes 4, 5), while treatment of infected cells with ceranib-2 and with SKI-II reduced the expression of rpS6 in comparison with infected untreated cells and in comparison with uninfected cells (Figure 2A). With respect to the phosphorylation of rpS6, we found that SKI-II caused a significant reduction at 16 and 24 h after infection (Figure 2A, lanes 8, 9 compared to 16, 17) indicating reduction of the translational capacity of the cells. There was a tendency of the MV infection to stimulate rpS6 phosphorylation. Especially, the intensity of the lower of the two phospho-rpS6 bands was increased at 16 and 24 h after infection (Figure 1, lanes 8, 9).

The expression of total eIF4E was not affected by the infection or inhibitor treatment (Figure 2B; evaluation not shown). However, eIF4E phosphorylation was increased at 16 h after infection and moderately decreased by ceranib-2 at 16 h and by SKI-II at 24 h after infection (Figure 2B).

The phosphorylation of MNK1 in comparison with total MNK1 protein was not affected (Figure 2C; evaluation not shown). The expression of total MNK1 was only slightly reduced by ceranib-2 treatment in infected cells (Figure 2C).

We also probed for IKK expression, since IKK and NF-κB were shown to be affected by SKI-II (Vijayan et al., 2014). The expression of IKK in MV-infected cells was reduced in the presence of SKI-II (Figure 2D). Thus, a decrease in total IKK-β levels would indirectly imply decreased NF-κB activity.

### Ceranib-2 and SKI-II Reduce MV Replication and Viral Protein Expression

**Figure 3:**
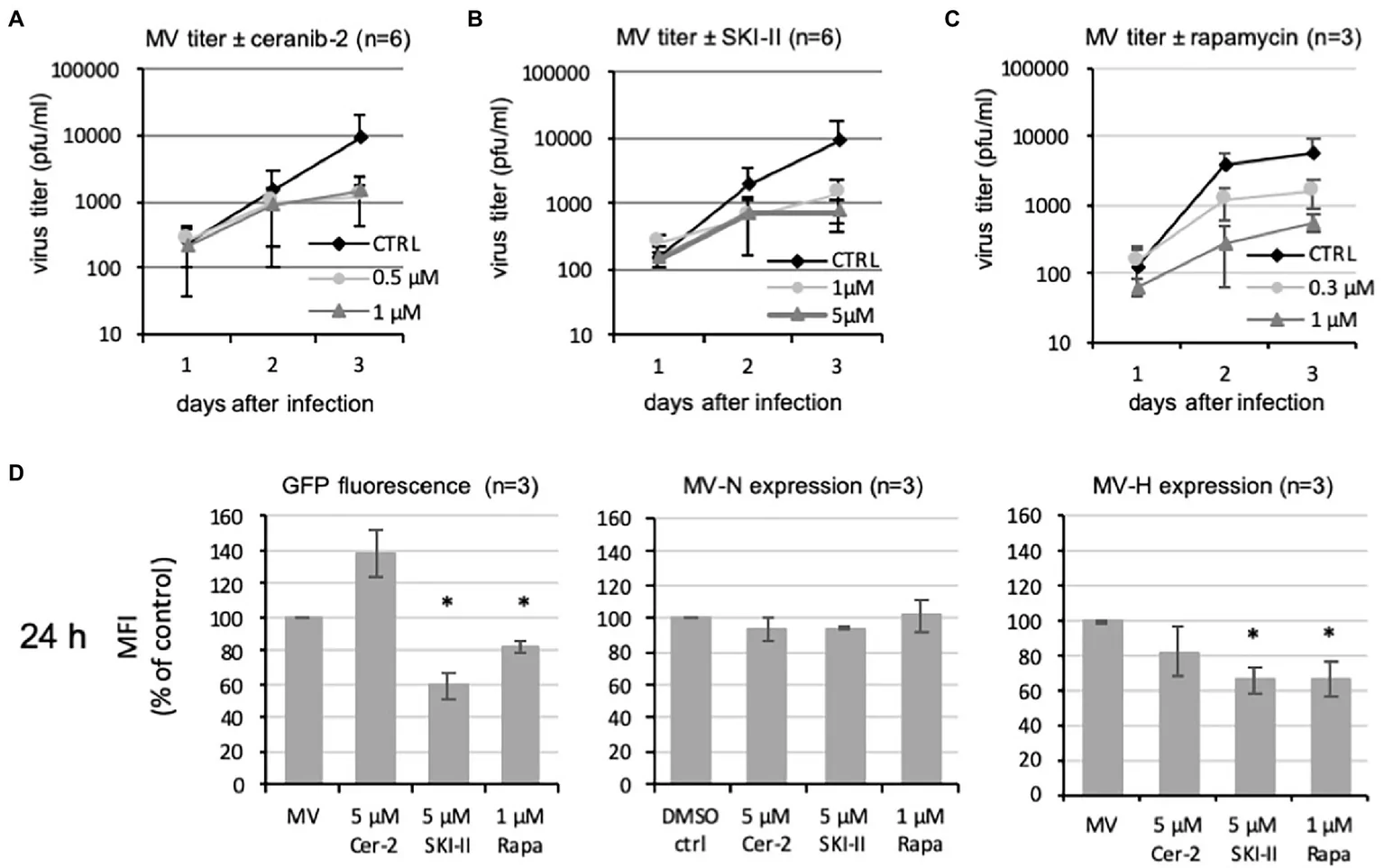
Effects of ceranib-2 and SKI-II on MV replication and viral protein expression in PBL. Primary human PBL were treated with 0.2% DMSO as mock control (CTRL) or 0.5 and 1 μM ceranib-2 **(A)**, 1 and 5 μM SKI-II **(B)** (data from Grafen et al., 2019 were reused), and 0.3 and 1 μM rapamyci **(C)** simultaneously to infection with MV (MOI = 0.1). Newly synthesized infectious virus (cell bound plus supernatant) was titrated using Vero-hSLAM cells 1, 2, and 3 days after infection and mean viral titers are shown (*n* = 6 for ceranib-2 and SKI-II and *n* = 3 for rapamycin). Reductions of viral titers were significant at days 2 and 3 (for clarity not shown in the graphic). In **(D)**, 24 h infected and inhibitor treated cells were analyzed by flow cytometry and the mean fluorescence intensities (MFI) of GFP, MV-nucleocapsid (MV-N), and MV-hemagglutinin (MV-H) in the cultures were quantified and presented as percent of control. Significant differences in comparison with the control were indicated with (*n* = 3; with ^*^p ≤ 0.05 calculated by student’s *t*-test).

Inhibitory effects by ceranib-2 and SKI-II on the production of infectious MV in PBL were observed at 48 and 72 h after infection. The viral titers were reduced by approximately one log step at 72 h after infection (Figures 3A,B). Recently, we demonstrated that efficient MV replication in PBL depends on mTORC1 activity and is inhibited by rapamycin (Tiwarekar et al., 2018). Also rapamycin led to approximately one log step reduced viral titers after 48 and 72 h of treatment (Figure 3C). Our findings that SKI-II and to a lesser extent ceranib-2 may affect eIF4E and rpS6 signaling suggest that this may affect the translational capacity of the cells and contribute to the reduction of MV replication.

We therefore investigated how ceranib-2 and SKI-II affect the levels of viral proteins. We took the virally encoded GFP as an easily and exactly quantifiable parameter for viral protein expression and analyzed its expression by flow cytometry. As expected, the mean fluorescence intensity (MFI) of GFP in SKI-II and rapamycin-treated cultures was reduced in comparison with control cultures at 24 h after infection (Figure 3D). However, the GFP fluorescence in ceranib-2-treated cultures was not reduced at 24 h but even has the tendency to increase. Thus, obviously ceranib-2 and SKI-II have differential effects on the GFP fluorescence. In addition, we quantified the expression the viral nucleocapsid protein (MV-N) and hemagglutinin (MV-H) using monoclonal antibodies by flow cytometry. The expression of MV-N at 24 h was not affected and MV-H was reduced by ceranib-2, SKI-II, and rapamycin (Figure 3D). This observation is consistent with a reducing effect of the inhibitors on the protein translation.

## Discussion

The two inhibitors ceranib-2 and SKI-II have been described as compounds with excellent potential for development as anticancer drugs (French et al., 2003; Draper et al., 2011; Santos and Lynch, 2015; Aurelio et al., 2016; Vethakanraj et al., 2018). In contrast to cancer cells, normal primary cells are less sensitive to these drugs and less prone to induction of apoptosis and cell death. This opens the possibility to apply these inhibitors as a supporting treatment against viral infections. SKI-II was described to have antiviral activity against influenza virus and MV (Seo et al., 2010; Vijayan et al., 2014; Grafen et al., 2019) and ceranib-2 against MV (Grafen et al., 2019). However, the mechanisms of antiviral action of both inhibitors are not clear, and it is not known if the inhibitors may act differentially or *via* common pathways. A mechanism including the inactivation of IKK and NF-kB has been suggested (Vijayan et al., 2014). We also observed an effect of SKI-II on the IKK expression (Figure 2D). We proposed additional interactions with mTORC1 and HSP90 (Grafen et al., 2019) and now investigated the effects of ceranib-2 and SKI-II on the mTORC1 downstream pathways which may affect the cellular translation. In uninfected cells, the major effect detected was a reduction of eIF4E phosphorylation by both inhibitors (Figure 1D).

We then observed that the MV infection led to increased rpS6 expression and a tendency to increased rpS6 phosphorylation (Figure 2A). This is consistent with our earlier finding that the MV infection induces a transient S1P increase which may stimulate the mTORC1 activity (Grafen et al., 2019). Furthermore, the rpS6 expression in infected cells was reduced by both inhibitors, while the phosphorylation of rpS6 was significantly reduced only by SKI-II. Thus, SKI-II had a stronger effect on rpS6 expression and phosphorylation than ceranib-2.

**Figure 4:**
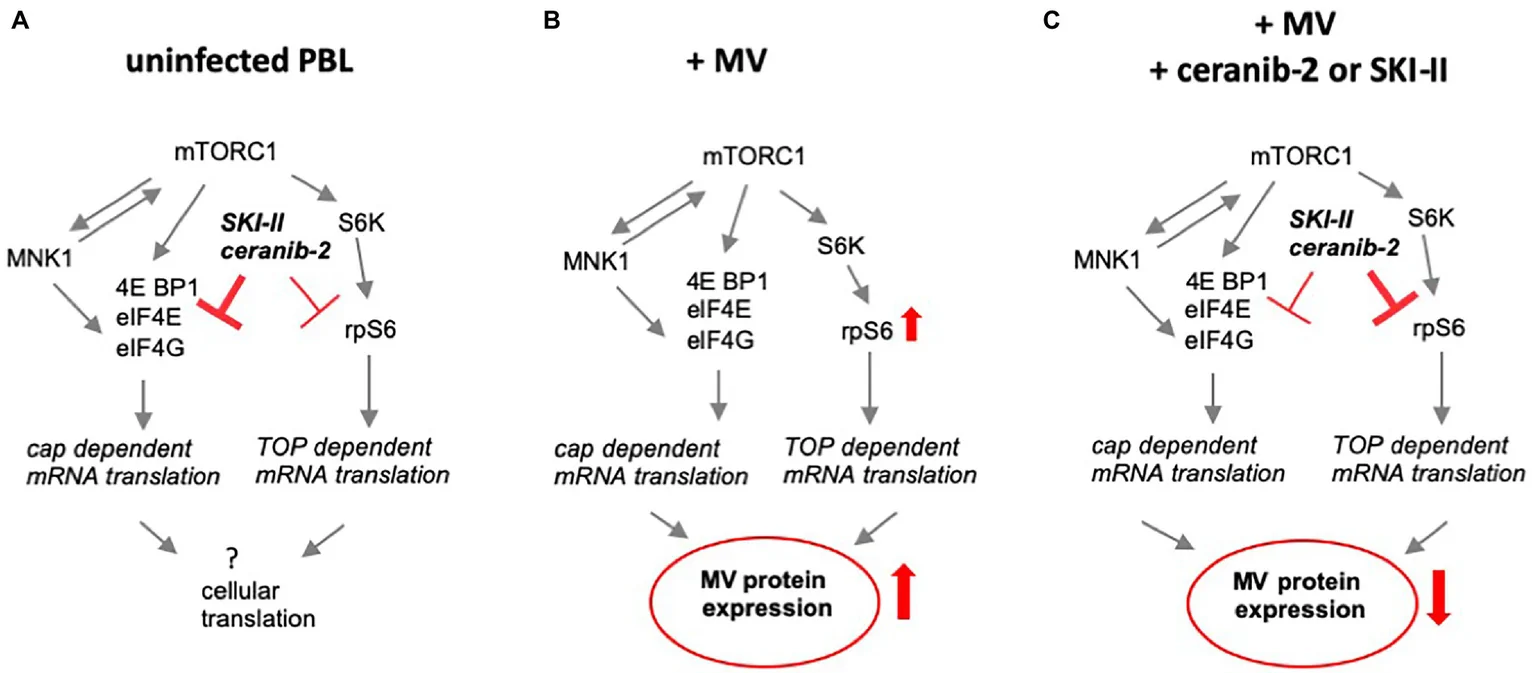
Schematic summary of the major findings. **(A)** Schematically summarizes the observed effects of ceranib-2 and SKI-II in uninfected PBL, **(B)** shows the effect of MV infection on these signaling pathways, and **(C)** the effects of the two inhibitors on infected PBL. Whereas in uninfected cells, the major effect was found on the phosphorylation of eIF4E, in MV-infected cells ceranib-2 and SKI-II predominantly neutralize the effect of the infection and reduce rpS6 expression below levels in untreated control cells.

The phosphorylation of eIF4E was only slightly reduced by both inhibitors (at 16 and 24 h) in comparison with infected cells, but not in comparison with uninfected cells (Figure 2B). In addition, SKI-II, but not ceranib-2, led to a reduction of the IKK expression (Figure 2D). Taken together, in MV-infected cells, SKI-II led to reductions of rpS6 and IKK, whereas ceranib-2 had only minor effects on the parameters tested (the findings were schematically summarized in Figure 4).

The synthesis of new infectious virus in PBL was reduced by ceranib-2 and SKI-II by approximately one log step (Figures 3A–C). This is similar to the result obtained with the mTORC1 inhibitor rapamycin, which suggested that similar mechanisms of viral inhibition might be involved. Analyzing the expression of viral proteins, we found that levels of MV-N and MV-H were reduced by ceranib-2, SKI-II, and rapamycin. This is consistent with and could be a consequence of reduced translational capacity of the cells, which may contribute to the observed reduction of the viral titers. However, when quantifying the viral GFP fluorescence, significant differences between the inhibitors were obvious. In contrast to the other inhibitors, there was a tendency that ceranib-2 led to increased levels of GFP fluorescence (Figure 3D). At the moment, we cannot explain this finding. There is a clear difference between the intracellular localization of acid ceramidase, which is present in lysosomes, and sphingosine kinases, which are found in the cytoplasm of nucleus of the cells. Thus, besides the described effects on mTORC1 signaling, ceranib-2 may affect other lysosomal functions, whereas SKI-II may also affect Hsp90 and NF-κB activities. Further experiments are required to clarify the involved mechanisms.

It is not clear if only the primary activity of the compounds (inhibition of acid ceramidase by ceranib-2 and inhibition of sphingosine kinases by SKI-II) mediated the observed effects, or if side effects may be responsible. SKI-II inhibits predominantly SphK2, but also SphK1 by inducing its degradation, and also dihydroceramide desaturase (Aurelio et al., 2016). In addition, it was found that SKI-II affects translation by interacting with membranes of the endoplasmic reticulum (ER), which activates the integrated stress response (ISR) and contributes to the toxicity of the inhibitor (Corman et al., 2021). These findings were supported by the observation that the compound is active also in cells lacking sphingosine kinases (Corman et al., 2021). Whether ceranib-2 may interact with ER or other membranes in similar ways is unknown.

We described earlier that ceranib-2 and SKI-II led to increased concentrations of ceramides of various chain lengths in BJAB cells and partially also in primary PBL. In addition, SKI-II reduced S1P concentrations in both cell cultures and prevented the transient increase of S1P between 0.5 and 6 h after infection as observed after MV infection (Grafen et al., 2019). Thus, sphingolipid-specific interactions may also contribute the observed effects of the inhibitors. To these specific effects belongs the signaling *via* TRAF2, RIP1, and Hsp90 to activate NF-κB (Xia et al., 2002; Alvarez et al., 2010; Park et al., 2015, 2016). It has been demonstrated that MV infection activates NF-κB in A549 cells early after infection (Helin et al., 2001), whereas the viral P and V proteins, which are newly synthesized after a lag phase of approximately 6 h after infection, suppress the activation of NF-κB in HEK293 cells (Schuhmann et al., 2011). Furthermore, inhibition of the NF-κB activation was demonstrated to inhibit MV replication in various cell lines (Vijayan et al., 2014). Here, we observed that IKK protein expression was reduced by SKI-II, which may lead to a reduced NF-κB activity, and thus is in accordance with these findings by Vijayan et al. (2014).

The antiviral activity of the acid ceramidase inhibitor ceranib-2 may predominantly be mediated *via* increasing concentrations of certain ceramides. A number of reports have described ceramide interacting proteins such as the anti-oncogene p53 (Fekry et al., 2018), ceramide activated protein kinase (Huwiler et al., 1996), PKCζ (Wang et al., 2005), cathepsins (Heinrich et al., 1999), and the ribosomal voltage-dependent anion channel 2 (Dadsena et al., 2019), which can induce apoptosis or other types of cell death. Predominantly C18 ceramide has been reported to interact with the SET (I2PP2A) protein and to inhibit its activity, which leads to an activation of the serine–threonine phosphatase PP2A, dephosphorylating, and inactivating the anti-apoptotic protein BCL2 (Dobrowsky et al., 1993; Li et al., 1996; Neviani et al., 2005; Trotta et al., 2007; Mukhopadhyay et al., 2009; Oaks and Ogretmen, 2014). Besides apoptosis, the PP2A complex consisting of three subunits regulates key cellular processes, such as cell-cycle progression, migration, and the cellular metabolism (Shi, 2009). As demonstrated for regulatory T cells, dephosphorylation by PP2A inactivates mTORC1 and thus reduces the cell metabolism including protein translation and lipid synthesis (Apostolidis et al., 2016).

In addition, PP2A also inhibits the activity of the mitogen-activated protein kinase interacting protein kinases 1 (MNK1; Li et al., 2010; Joshi and Platanias, 2014). Small interfering RNA-mediated knockdown of PP2A was found to result in increased phosphorylation of its direct target MNK1 and subsequently increased phosphorylation of eIF4E (Li et al., 2010). In our experiments, we found no effect of ceranib-2, SKI-II, or MV infection on the phosphorylation of MNK1.

In summary, our data indicate that ceranib-2, an inhibitor of the acid ceramidase, and SKI-II, an inhibitor of sphingosine kinases and dihydroceramide desaturase, differentially affect mTORC1 signaling *via* eIF4E and rpS6. In uninfected cells, the compounds predominantly reduce the eIF4E phosphorylation, whereas in MV-Infected cells, they affect the rpS6 expression. As a consequence of the reduced rpS6 levels, the translational capacity of the cells might be reduced and contribute to the antiviral effects observed after treatment of PBL with ceranib-2 and SKI-II.

## Data Availability Statement

The original contributions presented in the study are included in the article/supplementary material, further inquiries can be directed to the corresponding author.

## Author Contributions

JS-S: conceptualization. JC, HF, NL, and AG: methodology. JC and JS-S: writing-original draft preparation. All authors contributed to the article and approved the submitted version.

## Conflict of Interest

The authors declare that the research was conducted in the absence of any commercial or financial relationships that could be construed as a potential conflict of interest.

## Correction Note

A correction has been made to this article. Details can be found at: 10.3389/fphys.2026.1991227.

## Publisher’s Note

All claims expressed in this article are solely those of the authors and do not necessarily represent those of their affiliated organizations, or those of the publisher, the editors and the reviewers. Any product that may be evaluated in this article, or claim that may be made by its manufacturer, is not guaranteed or endorsed by the publisher.

## Abbreviations

5’TOP: 5′ terminal oligopyrimidine
ASM: acid sphingomyelinase
BCL2: B cell lymphoma-2
BJAB: Burkitt lymphoma B cell line
CAPK: cAMP-dependent protein kinase
DC-SIGN: dendritic cell-specific intercellular adhesion molecule-3 grabbing nonintegrin
eIF4E: eukaryotic initiation factor 4E
GAPDH: glycerinaldehyd-3-phosphat-dehydrogenase
GRP94: glucose regulating protein 94
Hsp90: heat shock protein 90
IKK: inhibitor of nuclear factor kappa-B kinase
ISR: integrated stress response
MNK1: mitogen-activated protein kinase interacting protein kinases 1
mTORC: mammalian target of rapamycin complex
MV: measles virus
MV-H: measles virus hemagglutinin
MV-N: measles virus nucleocapsid protein
NF-κB: nuclear factor kappa B
NOD: nucleotide-binding oligomerization domain-containing protein
PBL: peripheral blood lymphocytes
PHA: phytohemagglutinin
PKC: protein kinase C
PP2A: protein phosphatase 2A
RIP1/2: receptor (TNF receptor) interacting serine–threonine kinase 1/2
rpS6: ribosomal protein S6
S1P: sphingosine-1-phosphate
S6K: S6 protein kinase
SET: PP2A inhibitor
SKI-II: sphingosine kinase inhibitor 2
SphK: sphingosine kinase
SSPE: subacute sclerosing panencephalitis
TRAF2: TNF receptor-associated factor 2
VDAC2: voltage-dependent anion channel 2
