# Correction: The Sphingolipid Inhibitors Ceranib-2 and SKI-II Reduce Measles Virus Replication in Primary Human Lymphocytes: Effects on mTORC1 Downstream Signaling

**DOI:** 10.3389/fphys.2026.1991227

**Published:** 2026-10-14

**Authors:** Janice Chithelen, Hannah Franke, Nora Länder, Anika Grafen, Jürgen Schneider-Schaulies

**Affiliations:** Institute for Virology and Immunobiology, University of Würzburg, Würzburg, Germany

**Keywords:** acid ceramidase inhibitor ceranib-2, sphingosine kinase inhibitor SKI-II, mTORC1, translation, measles virus

There was a mistake in the caption of **Figure 1B** and **Figures 3A, B** as published. In these figure legends we did not indicate that data from a preceding publication were reused.

**Figure 1:**
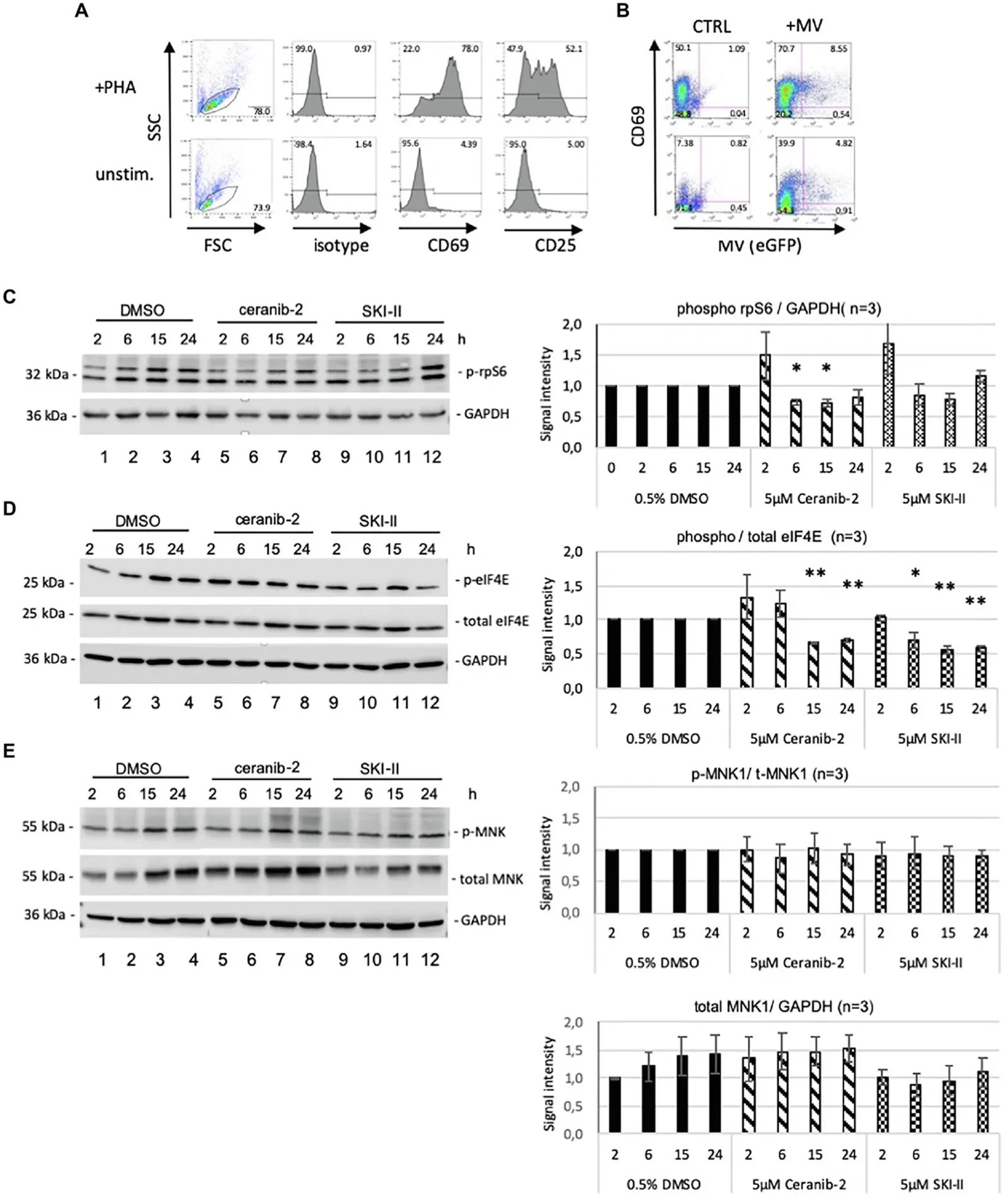
Effects of ceranib-2 and SKI-II on rpS6, eIF4E and MNK1 in PBL. In all experiments the quality of the PBL after PHA (2.5 µg/ml) stimulation was controlled by flow cytometry measuring the expression of CD69 and CD25 **(A)**, and the infection with MV (MV IC323eGFP) measuring the expression virally encoded GFP **(B)**. An example of infected and uninfected PBL in the presence and absence of PHA is shown representatively (data from Grafen et al., 2019 were reused).

**Figure 3:**
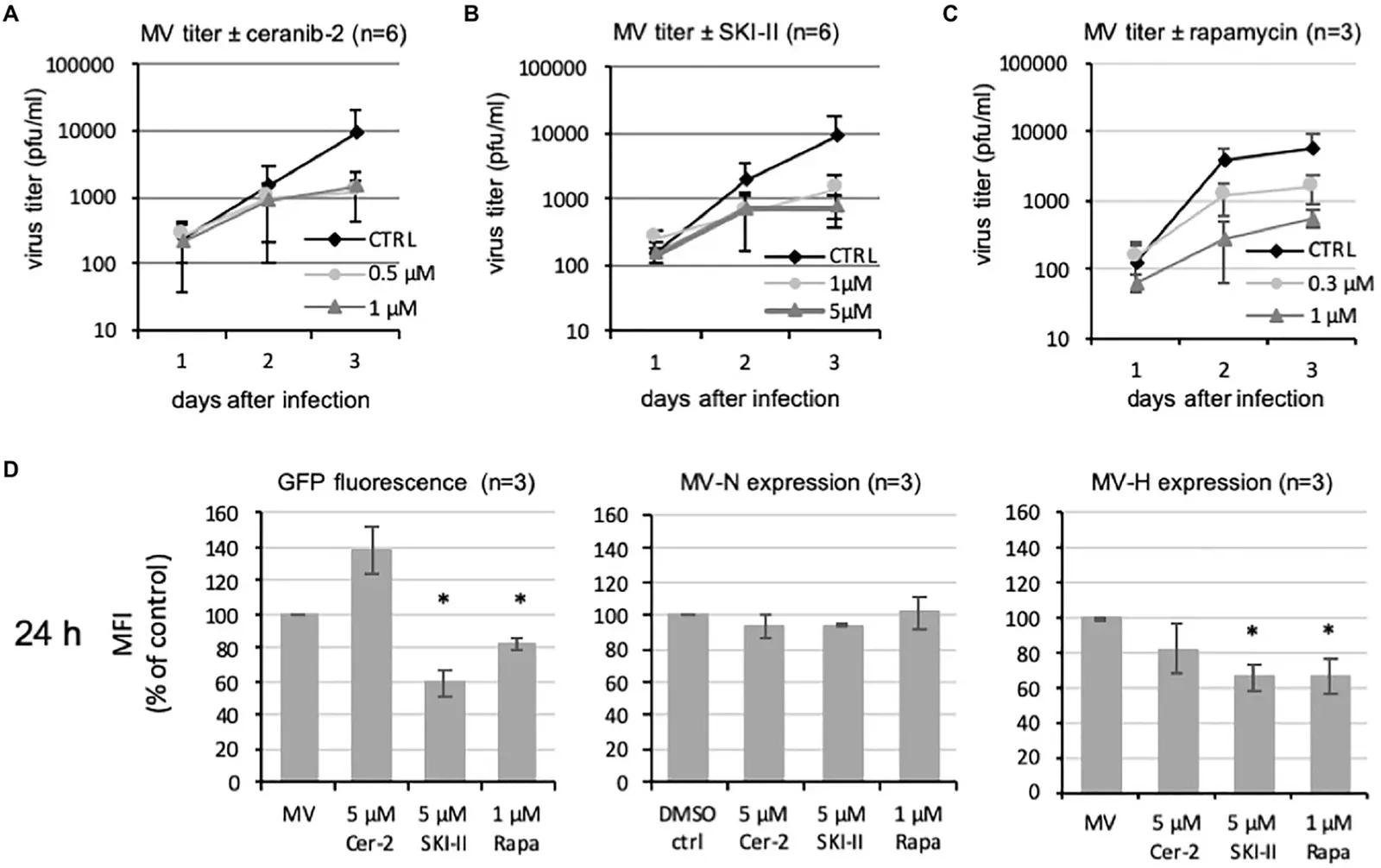
Effects of ceranib-2 and SKI-II on MV replication and viral protein expression in PBL. Primary human PBL were treated with 0.2% DMSO as mock control (CTRL) or 0.5 and 1 µM ceranib-2 **(A)**, 1 and 5 µM SKI-II **(B)** (data from Grafen et al., 2019 were reused), and 0.3 and 1 µM rapamycin **(C)** simultaneously to infection with MV (MOI = 0.1).

The corrected caption of **Figure 1B** and **Figures 3A, B** appears below.

The original version of this article has been updated.

